# Targeting mGlu Receptors for Optimization of Antipsychotic Activity and Disease-Modifying Effect in Schizophrenia

**DOI:** 10.3389/fpsyt.2019.00049

**Published:** 2019-02-14

**Authors:** Ferdinando Nicoletti, Rosamaria Orlando, Luisa Di Menna, Milena Cannella, Serena Notartomaso, Giada Mascio, Luisa Iacovelli, Francesco Matrisciano, Francesco Fazio, Filippo Caraci, Agata Copani, Giuseppe Battaglia, Valeria Bruno

**Affiliations:** ^1^Department of Physiology and Pharmacology, Sapienza University of Rome, Rome, Italy; ^2^IRCCS Neuromed, Pozzilli, Italy; ^3^Department of Psychiatry, The Psychiatric Institute, College of Medicine, University of Illinois, Chicago, IL, United States; ^4^Department of Drug Sciences, University of Catania, Catania, Italy; ^5^Oasi Research Institute (IRCCS), Troina, Italy; ^6^Institute of Biostructure and Bioimaging, National Research Council, Catania, Italy

**Keywords:** metabotropic glutamate receptors, schizophrenia, positive allosteric modulator, development of cortical interneurons, receptor cross-talk

## Abstract

Metabotropic glutamate (mGlu) receptors are considered as candidate drug targets for the treatment of schizophrenia. These receptors form a family of eight subtypes (mGlu1 to −8), of which mGlu1 and −5 are coupled to G_q/11_, and all other subtypes are coupled to G_i/o_. Here, we discuss the possibility that selective ligands of individual mGlu receptor subtypes may be effective in controlling the core symptoms of schizophrenia, and, in some cases, may impact mechanisms underlying the progression of the disorder. Recent evidence indicates that activation of mGlu1 receptors inhibits dopamine release in the meso-striatal system. Hence, selective positive allosteric modulators (PAMs) of mGlu1 receptors hold promise for the treatment of positive symptoms of schizophrenia. mGlu5 receptors are widely expressed in the CNS and regulate the activity of cells that are involved in the pathophysiology of schizophrenia, such as cortical GABAergic interneurons and microglial cells. mGlu5 receptor PAMs are under development for the treatment of schizophrenia and cater the potential to act as disease modifiers by restraining neuroinflammation. mGlu2 receptors have attracted considerable interest because they negatively modulate 5-HT_2A_ serotonin receptor signaling in the cerebral cortex. Both mGlu2 receptor PAMs and orthosteric mGlu2/3 receptor agonists display antipsychotic-like activity in animal models, and the latter drugs are inactive in mice lacking mGlu2 receptors. So far, mGlu3 receptors have been left apart as drug targets for schizophrenia. However, activation of mGlu3 receptors boosts mGlu5 receptor signaling, supports neuronal survival, and drives microglial cells toward an antiinflammatory phenotype. This strongly encourages research of mGlu3 receptors in schizophrenia. Finally, preclical studies suggest that mGlu4 receptors might be targeted by novel antipsychotic drugs, whereas studies of mGlu7 and mGlu8 receptors in animal models of psychosis are still at their infancy.

## Background

In spite of the continuous development of “monoaminergic” antipsychotic agents, drug treatment of schizophrenia remains suboptimal. Current second-generation antipsychotic drugs include drugs with different affinity for dopamine and serotonin receptor subtypes, such as clozapine, olanzapine, quetiapine, asenapine, ziprasidone, lurasidone, risperidone, paliperidone, iloperidone, aripiprazole, cariprazine, and brexpiprazole. These drugs display good therapeutic efficacy against positive symptoms of schizophrenia and their use may slow the progression of brain atrophy (as opposed to first generation antipsychotics) (1). However, a significant proportion of patients affected by schizophrenia is refractory to medication, and a prophylactic use of these agents (i.e., their use prior to the first episode of psychosis) is limited by class-related adverse effects, such as sedation, weight gain, and anticholinergic effects. In addition, none of the first- or second-generation antipsychotics is effective in improving cognitive dysfunction associated with schizophrenia, with the possible exception of clozapine, which is considered as a second- or third-line drug because of serious safety concerns.

Thus, there is an urgent need for new safer antipsychotic agents acting at new targets that lie at the core of the pathophysiology of schizophrenia. A new Molecular Psychiatry article entitled “mGluR5 hypofunction is integral to glutamatergic dysregulation in schizophrenia” (2) is one point of arrival of years of extensive research linking metabotropic glutamate (mGlu) receptors to the pathophysiology and treatment of schizophrenia. This article shows that mGlu5 receptor signaling is blunted in the dorsolateral prefrontal cortex (DLPFC) of individuals affected by schizophrenia (2).

mGlu5 is one of the eight mGlu receptor subtypes that are traditionally subdivided into three groups on the basis of their amino acid sequence, pharmacological profile, and signal transduction mechanisms in heterologous expression systems. Group I includes mGlu1 and mGlu5 receptors, which are coupled to G_q/11_ proteins. Group II (mGlu2 and mGlu3) and group III (mGlu4, mGlu6, mGlu7, and mGlu8) mGlu receptors are all coupled to Gi/o proteins (3). This classification is universally accepted but incomplete from a functional standpoint. Native mGlu receptors activate multiple transduction pathways in a cell-and context-dependent fashion, and numerous subtype-selective biased ligands have been developed. For example, mGlu5 receptors are physically and functionally linked to NMDA receptors in most of the CNS synapses, but there are biased-positive allosteric modulators (PAMs) of mGlu5 receptors that enhance mGlu5 receptor function without recruiting NMDA receptors (4, 5). There are also dogmas in the mGlu field that have been recently challenged. For example, mGlu3 receptors are generally considered as presynaptic receptors that function to restrain neurotransmitter release. In contrast, recent evidence indicates that mGlu3 receptors are also present in postsynaptic elements, where they boost mGlu5 receptor signaling (6). mGlu3 and mGlu5 receptors synergize to induce long-term depression (LTD) in the mouse PFC, and mGlu3-saturating LTD requires the activation of mGlu5 receptors (6). This receptor-receptor interaction may be relevant to the pathophysiology and treatment of schizophrenia (see below).

mGlu receptors were discovered in the mid eighties (7, 8), but no mGlu receptor ligands are currently available for clinical use in spite of more than 30 years of extensive research. In our opinion, schizophrenia and Parkinson's disease are the two disorders in which the mGlu research will first translate into the clinic. In section mGlu2 receptors and schizophrenia: to be or no to be? we will comment on a series of clinical studies in which pomaglumetad, the prodrug of the mGlu2/3 receptor agonist, LY404039, has been tested in patients affected by schizophrenia. Although pomaglumetad is no longer under clinical development, these studies attracted more and more interest on mGlu receptors in schizophrenia. It is generally believed that the activity of LY404039 in preclinical models and in subgroups of patients affected by schizophrenia is mediated by the activation of mGlu2 receptors. Here we will challenge the ≪ mGlu2-centric ≫ hypothesis of schizophrenia suggesting that mGlu3 receptors might be at least as valuable as mGlu2 receptors as condadate drug targets for schizophrenia. mGlu2 and mGlu3 receptors show important differences in the expression and function in the tripartite synapsis (pre- and post-synaptic elements, and astrocytes), and modulate the activity of microglial cells in an opposite fashion. We will also discuss the rationale behind the development of mGlu5 receptor PAMs and the attractive possibility that mGlu3 receptor agonists or PAMs might produce disease modifying effects in schizophrenia. Finally, we will comment on the attractive possibility that activation of mGlu1 receptors represents a valuable strategy in the treatment of positive symptoms of schizophrenia, and we will conclude with the discussion of a new set of evidence suggesting that mGlu4 receptors are also candidate drug targets for schizophrenia.

## Methods

We searched for the following terms on Pubmed: metabotropic glutamate receptors and schizophrenia, metabotropic glutamate receptors and psychosis, metabotropic glutamate receptors and neurodeveloment, metabotropic glutamate receptors and CNS development, metabotropic glutamate receptors and network activity.

## Targeting mGlu5 and mGlu3 Receptors to Correct Neurodevelopmental Alterations Associated With Schizophrenia

**Figure 1:**
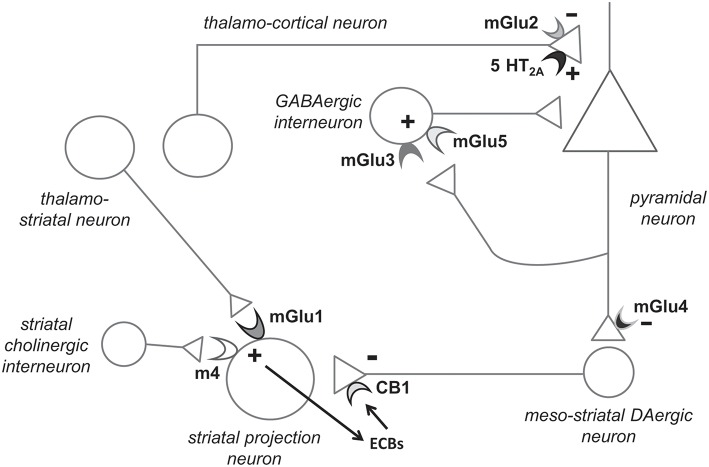
Hypothetical model highlighting the potential role of individual mGlu receptor subtypes in the pathophysiology and treatment of schizophrenia. Presynaptic mGlu2 receptors negatively modulate 5-HT_2A_ receptor signaling, and their activation restrains glutamate release at synapses between thalamo-cortical neurons and cortical pyramidal neurons (see chapter 6). mGlu5 receptors are found in both pyramidal neurons and interneurons (shown here) and functionally interact with NMDA receptors. mGlu5 receptor PAMs are under development for the treatment of schizophrenia (see chapter 4). Recent evidence indicates that mGlu3 receptor activation boost mGlu5 receptor signaling, and, therefore, selective mGlu3 receptor agonists or PAMs should be tested in preclinical models predictive of antipsychotic activity (chapter 3). The functional cross-talk between mGlu3 and mGlu5 receptors has been shown in pyramidal neurons (6). We speculate that the two receptors also interact in GABAergic interneurons, but this remains to be demonstrated. mGlu1 receptors and type-4 muscarinic cholinergic receptors (M4) interact in stimulating the production of endocannabinoids (ECBs) in striatal projection neurons. ECBs activate presynaptic CB1 receptors, thereby reducing dopamine (DA) release from meso-striatal terminals. Hence, mGlu1 receptor PAMs might be effective in improving positive symptoms in schizophrenia (chapter 5). Finally, mGlu4 receptor agonists/PAMs are effective in animal models that are predictive of antipsychotic activity. mGlu4 receptors might act to reduce glutamate release from excitatory nerve endings of pyramidal neurons and through other mechanisms. Not shown here: activation of mGlu3 and mGlu5 receptors may restrain neuroinflammation by driving microglia toward an antinflammatory phenotype, and activation of mGlu3 receptors stimulates the production of neurotrophic factors in astrocytes (see chapters 3 and 4).

A large body of evidence suggests that schizophrenia is a neurodevelopmental disorder in which cortical interneurons become dysfunctional as a result of genetic alterations or environmental challenges occurring in the perinatal period. There are at least four processes that characterize the development of cortical GABAergic interneurons: (i) the biochemical specification into different cell types, of which those containing parvalbumin (PV) or somatostatin (SSt) are the most numerous; (ii) the correct matching between interneurons and pyramidal neurons (9); (iii) the GABA shift from excitatory into inhibitory driven by the expression of the potassium-chloride symport, KCC2 in mature neurons (10); and (iv) the formation of perineuronal nets (PNNs), which surround PV^+^ interneurons at the offset of the critical temporal windows of developmental plasticity (11, 12). The molecular events that drive these processes are only partially elucidated. Schizophrenia is associated with a dysfunction of PV^+^ (basket and chandelier) and other populations of interneurons, with a resulting defect in network oscillations underlying cognitive functions (13) (Figure 1). In addition, several lines of evidence suggest that formation of PNNs is altered in the PFC of patients affected by schizophrenia (14–16). What may link mGlu3 and mGlu5 receptors to these processes is the evidence that the expression of both receptors is high in the early postnatal development, and then progressively declines to reach adult levels after weaning (17). One of the first observations associated with the discovery of mGlu receptors was that mGlu receptor-mediated polyphosphoinositide (PI) hydrolysis (a biochemical process leading to intracellular Ca^2+^ mobilization and activation of protein kinase C) is dramatically high in the cerebral cortex and other brain regions during the early 7–9 days of postnatal life, and declines afterwards (8). This robust PI response is mediated by mGlu5 receptors, which are heavily expressed early after birth even in cells that lack mGlu5 receptors in the adult life, such as cerebellar Purkinje cells (18). An unexpected finding was that a large component of mGlu5-mediated PI hydrolysis in the developing PFC was lost in mGlu3^−/−^ mice, and, therefore, required the endogenous activation of mGlu3 receptors (6). In contrast, mGlu5 receptor-mediated PI hydrolysis was intact in mGlu2^−/−^ mice (6). Interestingly, expression of mGlu2 receptors in the cerebral cortex is low in the early postnatal life and increases afterwards (as opposed to expression of mGlu3 and mGlu5 receptors) (17).

Which (if any) of the neurodevelopmental processes taking place early after birth is/are driven by the combined activation of mGlu3 and mGlu5 receptors? This question is starting to be investigated. A reduction in the transcript and protein levels of PV, SSt, GAD-65, GAD-67, Reelin, and AMPA and NMDA receptor subunits has been reported in the PFC of adult mGlu5^−/−^ mice (19). In mGlu3^−/−^ mice we found changes in biochemical markers of cortical GABAergic interneurons during the first 4 weeks of postnatal development, which were not seen in mGlu2^−/−^ mice (20). These findings suggest that the functional partnership between mGlu3 and mGlu5 receptors may be instrumental for the biochemical and functional specialization of cortical GABAergic interneurons. An attractive hypothesis is that the two receptors may also serve to regulate the numeric balance between pyramidal neurons and GABAergic interneurons in the developing cerebral cortex. An elegant article by Prof. Oscar Marin and his Associates (9) demonstrates that the balanced network between excitatory and inhibitory cortical neurons becomes established through mechanisms of activity-dependent cell death and survival, with the decrease in the total number of pyramidal neurons preceding the decrease in the number of interneurons in the first few days of postnatal life. Surviving interneurons are those that are innervated by pyramidal cells because synaptic excitation enhances the activity of the phosphatidylinositol-3-kinase (PI3K) pathway by suppressing the expression of the PI3K negative regulator, phosphatase and tensin homolog PTEN (9). Both mGlu3 and mGlu5 receptors may signal through the activation of the PI3K pathway (3), and we are currently examining whether genetic and pharmacological manipulation of the two receptors alters the expression of total and phosphorylated PTEN in the developing cerebral cortex. Another interesting area of investigation is whether mGlu5 and mGlu3 receptors regulate KCC2 expression and GABA responses in pyramidal cells during postnatal development. Changes in cortical and cerebellar KCC2 levels were found in mice lacking mGlu3 and mGlu5 receptors, respectively (20, 21).

How all this can be relevant to the pathophysiology and experimental treatment of schizophrenia? Cell-specific deletion of mGlu5 receptors in PV^+^ neurons in mice results into a behavioral phenotype modeling schizophrenia, characterized by spontaneous hyperactivity, and defects in pre-pulse inhibition and long-term memory (22). A psychotic-like phenotype was also observed in mice lacking mGlu3 receptors (23, 24). Wang et al. (2) found that mGlu5 receptor signaling was largely reduced in the post-mortem DLPFC of individuals affected by schizophrenia, as shown by tyrosine hyperphosphorylation (leading to receptor desensitization), reductions in receptor coupling to G_q/11_, Homer, and PI3K, and reduced receptor association with adaptor and scaffolding proteins, such as RGS4, norbin, Preso 1 and tamalin. Remarkably, the reduced signaling of mGlu5 receptors disrupted the physiological interplay between mGlu5 and NMDA receptors in the DLPFC of individuals affected by schizophrenia (2). Abnormalities in mGlu3 receptor dimerization have also been reported in the frontal cortex of individuals affected by schizophrenia (25). Polymorphic variants of GRM3 (the gene encoding for the mGlu3 receptor) have been consistently associated with schizophrenia (26–37), and genetic alterations of GRM5 have also been linked to schizophrenia (38).

We hypothesize that, in schizophrenia, a reduced expression and/or activity of mGlu3 and mGlu5 receptors may have a strong impact on the maturation of cortical GABAergic interneurons leading to a permanent alteration of network activity in the frontal cortex. If turned to be correct, this hypothesis raises the interesting possibility that a pharmacological intervention with mGlu3 or mGlu5 receptor PAMs early after birth may restore the correct developmental trajectory rescuing the pathological phenotype. This is a testable hypothesis in animal models, but translation to humans is an extremely difficult task for ethical and practical reasons. Regardless of any potential therapeutical application, defining the precise role of mGlu3 and mGlu5 receptors in neuronal development will give an answer to an intriguing question raised since the discovery of mGlu receptors: why mGlu receptor-mediated PI hydrolysis is so large in the early postnatal life (much larger than PI hydrolysis mediated by any other receptor coupled to G_q/11_) and progressively declines afterwards?

Another potential link between mGlu5 receptor signaling and schizophrenia is suggested by the evidence that the endogenous D-amino acid, D-aspartate, has a great efficacy in stimulating PI hydrolysis in cortical or hippocampal slices prepared from 8-9 day-old rats, and its action is largely mediated by mGlu5 receptors (39). D-Aspartate levels are reduced in the dorsolateral prefrontal cortex of patients affected by schizophrenia as a result of an enhanced activity of the catabolic enzyme, D-aspartate oxidase (40, 41). In addition, the antipsychotic drug, olanzapine, enhances glutamate release in the prefrontal cortex by inhibiting D-aspartate catabolism (42). Thus, a defective D-aspartate/mGlu5 receptor axis might contribute to the pathophysiology of schizophrenia, and some antipsychotic drugs might reinforce mGlu5 receptor signaling by enhancing endogenous D-aspartate levels. Whether D-aspartate directly activates mGlu5 receptors, or receptor activation is secondary to the enhanced glutamate release induced by D-aspartate remains to be determined.

## mGlu5 Receptor PAMs as Novel Antipsychotic Agents

The leading hypothesis of NMDA receptor hypofunction in schizophrenia stems from the strong psychotomimetic effects of slow NMDA receptor channel blockers, such as phenyclidine (PCP) and ketamine, the cognitive dysfunction and psychosis caused by NMDA receptor autoantibodies in patients affected by autoimmune encephalitis and the large body of evidence obtained in mice lacking NMDA receptors or treated with NMDA receptor antagonists. NMDA receptors are found in most CNS neurons; however, they are highly expressed and functional in cortical interneurons (43). Several drugs that activate NMDA receptors have been developed for the treatment of schizophrenia, but with limited success in clinical trials. One notable example is bitopertin, a drug that inhibits the high affinity uptake of the NMDA receptor co-agonist, glycine. After promising preclinical and phase-2 clinical studies, bitopertin failed in phase-3 trials (44). Although clinical data with bitopertin were disappointing, active work on NMDA receptor agonists continues. A functional cross-talk exists between mGlu5 and NMDA receptors. Activation of mGlu5 receptors facilitates NMDA receptor function (45–50), whereas activation of NMDA receptors amplifies mGlu5 receptor activity by restraining receptor desensitization (51). This and other observations laid the groundwork for the development of mGlu5 reptor PAMs for the treatment of schizophrenia. Jeff Conn, Carrie Jones, Craig Lidsley and their Associates (Vanderbilt University) are actively involved in the design and development of highly selective and brain permeant mGlu5 receptor PAMs, and they are so kind to make their molecules available to the scientific community. PAMs differ from orthosteric agonists because they have no intrinsic efficacy but amplify the action of endogenous glutamate. Thus, PAMs are particularly advantageous from a therapeutical standpoint because their action is activity-dependent. Systemic treatment with mGlu5 PAMs corrects the pathological phenotype in pharmacological and genetic models that have predictive validity for antipsychotic treatment, including models of NMDA receptor hypofunction (52–58) (Figure 1). Excitotoxicity mediated by the enhanced NMDA receptor activity is a potential limtation to the use of mGlu5 receptor PAMs in the treatment of schizophrenia. Accordingly, high doses of mGlu5 receptor PAMs may cause seizures and neurotoxicity in rodents (4, 59, 60). An elegant way to overcome these limitations is the use of biased mGlu5 receptor PAMs that amplify receptor function without recruiting NMDA receptors (4, 5). One of these molecules, compound VU0409551, showed antipsychotic-like activity without activating NMDA receptors (5).

Biased mGlu5 PAMs might be particularly helpful in the treatment of patients affected by schizophrenia who are resistant to conventional antipsychotic medication. Our expectation is that these drugs may improve both positive and negative symptoms and exert pro-cognitive effects by restoring the balance between excitation and inhibition in the prefrontal cortex of patients affected by schizophrenia (58). While an early treatment with mGlu5 PAMs might correct the developmental abnormalities associated with schizophrenia (see above), the impact of a late treatment on disease progression cannot be predicted. A large body of evidence suggests that neuroinflammation has a key role in the progressive degeneration of the gray and white matter associated with schizophrenia (61–63). Interestingly, mGlu5 receptors are present in microglial cells, and their activation drives microglia toward an anti-inflammatory phenotype (64–69).

A potential anti-inflammatory effect of mGlu5 PAMs may slow disease progression in the absence of a direct induction of excitotoxic neuronal death. Thus, biased PAMs that enhance mGlu5 receptor activation without potentiating NMDA receptor currents cater the potential to behave as disease modifyiers in schizophrenia. This interesting hypothesis warrants further investigation in animal models of psychosis associated with neuroinflammation.

## Targeting mGlu1 Receptors in the Treatment of Psychosis

An elegant manuscript describes a novel form of receptor-receptor interaction involving M4 muscarinic acetylcholine receptors and mGlu1 receptors (70). This interaction might pave the way to novel therapeutic strategies in schizophrenia. M4 receptor activation inhibits striatal dopamine release by enhancing the production of endocannabinoids, which behave as retrograde messengers at dopaminergic nerve terminals. Interestingly, this mechanism requires the co-activation of mGlu1 receptors, which are coupled to G_q/11_, and, therefore, have the *fisique du role* to enhance the production of endocannabinoids. M4 receptors, which are coupled to G_i/o_, are also able to inhibit D1 receptor signaling, but this function is independent of mGlu1 receptors (70) (Figure 1). Inhibition of dopamine release mediated by mGlu1 receptors explains the antipsychotic-like activity of selective mGlu1 PAMs in rodents (70) and holds promise for the treatment of positive symptoms of schizophrenia. Mutations of GRM1 (the gene encoding for the mGlu1 receptor) that reduce mGlu1 receptor signaling have been associated with schizophrenia (71), and mice lacking mGlu1 receptors display a psychotic-like phenotype (72). This suggests that a defective mGlu1 receptor expression and/or activity might contribute to the striatal dopaminergic hyperactivity associated with schizophrenia, and that mGlu1 receptor PAMs may correct this defect. Of note, co-activation of M4 and mGlu1 receptors selectively inhibits dopamine release in the striatum without affecting dopaminergic transmission in other brain regions. This suggests that mGlu1 receptor PAMs may reduce dopamine release only *where is needed*, i.e., in the hyperactive meso-striatal system. This is advantageous with respect to most antisychotic agents, which block D2 receptors with no regional discrimination.

The interplay between mGlu1 and M4 receptors is one of the many examples of functional interactions between G_q/11_-coupled group-I mGlu receptors and other G_i/o_ coupled receptors. In the cerebellum, GABA_B_ receptors cooperate with mGlu1 receptors at the synapses between parallel fibers and Purkinje cells (73). Andrzej Pilc and his Associates (Krakow University, Polland) have described a synergistic effect between mGlu5 and GABA_B_ receptor PAMs in behavioral tests with pharmacological validity for the treatment of positive, negative and cognitive symptoms of schizophrenia (74).

## mGlu2 Receptors and Schizophrenia: to be or no to be?

The study of mGlu2 receptors as druggable targets in schizophrenia has been a leading theme of research in the whole mGlu receptor field in the last two decades. The evidence that compound LY354740 (a potent, selective, and brain permeant mGlu2/3 agonist) was able to reverse behavioral and neurochemical effects of PCP in rats (75) gave the impetus to the development of orthosteric mGlu2/3 receptor agonists or mGlu2 receptor PAMs as novel “non-monoaminergic” antipsychotic agents. mGlu2 receptors are presynaptic receptors that inhibit neurotransmitter release (3). It is believed that mGlu2 receptor activation produces antipsychotic effects by restraining the activity of 5-HT_2A_ receptors in the frontal cortex. Gerard Marek and his Associates were first to demonstrate that mGlu2 receptors inhibit electrophysiological responses mediated by 5-HT_2A_ receptors (76, 77). In the following years, Javier Gonzalez-Maeso and his Associates showed that mGlu2 receptors form a multimeric complex with 5-HT_2A_ receptors and negatively modulate 5-HT_2A_ receptor signaling in response to serotonin-like hallucinogenic drugs (78–81). Expression of mGlu2 receptors in the mouse frontal cortex is under the control of 5-HT_2A_ receptors, and repeated administrations of second-generation antipsychotics, which block 5-HT_2A_ receptors, down-regulates the expression of mGlu2 receptors through an epigenetic mechanism mediated by reduced histone acetylation at the GRM2 gene promoter (82, 83). Interestingly, mGlu2 receptors are down-regulated in the frontal cortex of mice subjected to prenatal stress (84, 85), which models the epigenetic and behavioral modifications associated with schizophrenia (84, 86). Not surprisingly, both mGlu2/3 agonists and mGlu2 PAMs display robust antipsychotic-like activity in a variety of behavioral tests (87–90).

The clinical development of the mGlu2/3 agonist, LY404039 (under the form of the oral prodrug, pomaglumetad methionil) is a remarkable example of how translational research in schizophrenia is conditioned by a number of unpredictable variables. Pomaglumetad methionil was as efficacious as olanzapine in improving positive and negative symptoms of schizophrenia in the first phase 2 clinical trial (91) (Figure 1). Treatment with pomaglumetad methionil did not cause the typical adverse effects of antipsychotic drugs, such as extrapyramidal symptoms, increase in body weight, and hyperprolactinemia (91). These findings generated great enthusiasm, demonstrating for the first time the efficacy of a non-monoaminergic agent in the treatment of schizophrenia. Unfortunately, the antipsychotic activity of pomaglumetad methionil was not confirmed in subsequent phase 2 or 3 clinical trials (92–94), and this led to discontinuation of the development program of LY404039 in schizophrenia with negative consequences for the whole mGlu receptor field. However, a metanalysis of all clinical studies showed that pomeglumetad methionil displayed therapeutic efficacy in subgroups of patients who were early-in-disease and had never been treated with 5-HT_2A_ blocking agents (95). Research on the effect of LY404039 in humans is still ongoing, and both pomaglumetad methionil and LY2979165 (the oral prodrug of the selective mGlu2 receptor agonist, 2812223) were found to reduce ketamine-evoked blood oxygenation level dependent (BOLD) MRI signal in healthy subjects (96). It will be interesting to design clinical trials in which mGlu2/3 receptor agonists (or mGlu2 receptor PAMs) are tested in selected populations of patients affected by schizophrenia recruited on the basis of their genetic background, disease course, and history of previous medication. We are in favor of the development of mixed mGlu2/3 agonists or PAMs (rather than selective mGlu2 PAMs) if we consider the potential impact of all these drugs on disease progression. We have evidence that selective activation of mGlu2 receptors may produce neurotoxic effect (97–99) by enhancing the proinflammatory activity of microglia (100–102) or through other mechanisms. In contrast, activation of mGlu3 receptors is consistently neuroprotective through a mechanism of astrocyte-neuronal interaction mediated by the production of neurotrophic factors, such as transforming-growth factor-β1 or glial cell-derived neurotrophic factor (98, 103–105).

## mGlu4 Receptors as Novel Targets for the Treatment of Schizophrenia

The study of individual group-III mGlu receptor subtypes in schizophrenia is now facilitated by the availability of brain permeant subtype-selective agents that are suitable for *in vivo* studies. One of these drugs is compound LSP4-2022, which behaves as a preferential orthosteric agonist of mGlu4 receptors (106). Systemic treatment with LSP4-2022 has been shown to induce antipsychotic-like activity in a number of behavioral tests and to attenuate neurotransmitter release induced by the NMDA receptor antagonist, MK-801 (107, 108). Interestingly, mGlu4 receptors co-operate with other neurotransmitter receptors coupled to G_i/o_ in improving psychotic-like symptoms in mice. Wozniak et al. (108) showed that the antipsychotic-like activity of LSP4-2022 in mice was prevented by pharmacological blockade of 5-HT_1A_ receptors, whereas sub-threshold doses of LSP4-2022 and the 5-HT_1A_ agonist, 8-hydroxy-dipropylaminotetraline (8-OH-DPAT), acted synergistically in producing antipsychotic- like effects. A similar synergism was shown between LSP4-2022 and drugs that activate GABA_B_ receptors, although in this case the synergism could only be demonstrated in behavioral tests that model positive symptoms of schizophrenia (107).

A more recent study exended the analysis to the interaction between mGlu4 and M4 muscarinic receptors in an attempt to develop novel pharmacological strategies with good efficacy in improving positive, negative and cognitive symptoms, and good profile of safety and tolerability. A robust effect was seen by combining subactive doses of LSP4-2022 and the selective M4 muscarinic receptor PAM, VU152100, in behavioral tests that model positive, negative, and cognitive symptoms. This combination also reduced 5-HT_2A_-mediated spontaneous excitatory postsynaptic currents in frontal cortical slices, indicating that mGlu4 and M4 receptors cooperate in reducing glutamate release (109). Remarkably, the association of subactive doses of drugs that activate mGlu4 and M4 receptors did not cause motor impairment in mice, suggesting that the functional cross-talk between mGlu4 and M4 receptors can be targeted by new safer antipsychotic agents (Figure 1).

Finally, the study of mGlu4 receptors provides a new potential link beween the kynurenine pathway and schizophrenia. The kynurenine pathway of tryptophan metabolism generates a series of neuroactive componds, including the mGlu4 receptor agonist, cinnabarinic acid (110). We have evidence that cinnabarinic acid inhibits behavioral and biochemical responses to MK-801 at very low doses (111). Cinnabarinic acid is synthesized in very low amounts in the normal brain, but its production increases considerably under conditions of neuroinflammation. A protective activity of endogenous cinnabarinic acid against neuroinflammation (112) and psychotic symptoms (113) might be lost in schizophrenia because of a lower activity of kynurenine monoxygenase (114–116), the enzyme that converts kynurenine into 3-hydroxykynurenine giving rise to all metabolites that lie downstream of 3-hydroxykynurenine. This is certainly a field of great interest that warrants further investigation.

## Conclusions

Subtype-selective mGlu receptor ligands offer the opportunity of a precision medicine based pharmacological approach to target multiple domains of the psychopathological spectrum of schizophrenia. For example, patients with mutations of GRM1 or GRM3 genes might benefit from a treatment with mGlu1 and mGlu3 receptor PAMs, respectively, whereas patients who are early in disease or had never been treated with second-generation antipsychotics might respond to mGlu2/3 receptor agonists. The increasing knowledge of the role played by mGlu3 and mGlu5 receptors in the developmental trajectory of GABAergic interneurons might pave the way to an early use of mGlu3 or mGlu5 receptor PAMs as disease modifying agents, being aware that treatment of schizophrenia in the preclinical phase awaits the discovery of predictive biomarkers endowed with high specificity and selectivity. Finally, some mGlu receptor ligands might have a positive impact on neuroinflammation by influencing microglial function or through other mechanisms. There are no reasons to be skeptical and continue to develop new mGlu receptor ligands for the treatment of schizophrenia with good hope of success.

## Author Contributions

All authors listed have made a substantial, direct and intellectual contribution to the work, and approved it for publication.

### Conflict of Interest Statement

The authors declare that the research was conducted in the absence of any commercial or financial relationships that could be construed as a potential conflict of interest.
